# High‐gamma oscillations precede visual steady‐state responses: A human electrocorticography study

**DOI:** 10.1002/hbm.25196

**Published:** 2020-09-04

**Authors:** Benjamin Wittevrongel, Elvira Khachatryan, Evelien Carrette, Paul Boon, Alfred Meurs, Dirk Van Roost, Marc M. Van Hulle

**Affiliations:** ^1^ Laboratory for Neuro‐ and Psychophysiology KU Leuven Leuven Belgium; ^2^ Laboratory of Clinical and Experimental Neurophysiology Ghent University Hospital Ghent Belgium; ^3^ Department of Neurosurgery Ghent University Hospital Ghent Belgium

**Keywords:** cross‐frequency coupling (CFC), electrocorticography (ECoG), frequency tagging, phase locking, phase‐amplitude coupling (PAC), photic driving, SSVEP

## Abstract

The robust steady‐state cortical activation elicited by flickering visual stimulation has been exploited by a wide range of scientific studies. As the fundamental neural response inherits the spectral properties of the gazed flickering, the paradigm has been used to chart cortical characteristics and their relation to pathologies. However, despite its widespread adoption, the underlying neural mechanisms are not well understood. Here, we show that the fundamental response is preceded by high‐gamma (55–125 Hz) oscillations which are also synchronised to the gazed frequency. Using a subdural recording of the primary and associative visual cortices of one human subject, we demonstrate that the latencies of the high‐gamma and fundamental components are highly correlated on a single‐trial basis albeit that the latter is consistently delayed by approximately 55 ms. These results corroborate previous reports that top‐down feedback projections are involved in the generation of the fundamental response, but, in addition, we show that trial‐to‐trial variability in fundamental latency is paralleled by a highly similar variability in high‐gamma latency. Pathology‐ or paradigm‐induced alterations in steady‐state responses could thus originate either from deviating visual gamma responses or from aberrations in the neural feedback mechanism. Experiments designed to tease apart the two processes are expected to provide deeper insights into the studied paradigm.

## Graphical abstract

The robust steady‐state cortical activation elicited by flickering visual stimulation has been exploited by a wide range of neuroscientific and clinical studies, but the neural mechanisms behind its generation are not well understood. In this manuscript, we show that the fundamental response is preceded by high‐gamma (55–125 Hz) oscillations which are also synchronised to the gazed frequency. Using a subdural recording of the primary and associative visual cortices of one human subject, we demonstrate that both latencies of the gamma and fundamental components are highly correlated on a single‐trial basis albeit that the latter is consistently delayed by approximately 55 ms.

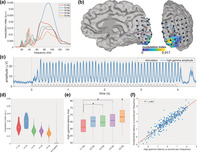

## 1 INTRODUCTION

From the earliest studies on the visual system (Adrian & Matthews, 1934; Walter, Dovey, & Shipton, 1946), flickering stimulation has routinely been adopted to elicit robust neural activations using either on–off or luminosity modulations with a checkerboard (Krolak‐Salmon et al., 2003), gratings (Kim, Grabowecky, Paller, Muthu, & Suzuki, 2007), or plain rectangles (Lee, Pokorny, Smith, & Kremers, 1994). The evoked neural response, which inherits the spectral properties of the stimulation, is often referred to as the “steady‐state visual evoked potential” (SSVEP) or, in clinical studies, as “photic driving” (Regan, 1966; van der Tweel & Lunel, 1965). Its high signal‐to‐noise ratio and low sensitivity to ocular artefacts render it a robust response that is relatively easy to detect in electrophysiological settings (Vialatte, Maurice, Dauwels, & Cichocki, 2010). Flickering stimulation is also routinely used in combination with hemodynamic recording modalities (e.g., functional magnetic resonance imaging [fMRI]) where the neural response is reflected in increased BOLD responses which have formed the basis for the development of a non‐invasive retinotopic mapping procedure of visual cortical areas (Engel, Glover, & Wandell, 1997) and even lateral geniculate nucleus (LGN) (Chen, Zhu, Thulborn, & Ugurbil, 1999). It is worth noting that the steady‐state response elicited by flickering stimuli not only contributed to advances in basic research, but also many clinical studies have used the properties (e.g., amplitude, latency) of the automatic cortical response as a marker for the assessment of neurological or psychiatric conditions (Vialatte et al., 2010), and as the basis for the most efficient brain‐computer interfaces (Chen et al., 2015; Wittevrongel, Khachatryan, Fahimi Hnazaee, Camarrone, et al., 2018) aimed to support communication‐impaired patients (Combaz et al., 2013; Hwang et al., 2017; Lesenfants et al., 2014).

However, despite its widespread adoption, relatively little is known about the cortical mechanisms involved in the processing of the repetitive visual input and the origin of the fundamental steady‐state response. While there is general consensus that this response mainly originates in the primary visual cortex, the neural mechanisms behind its genesis are still unknown: it is not clear whether it is a pure sensory response or whether larger cortico‐cortical and/or cortico‐thalamic networks contribute to it (Vialatte et al., 2010). Studying the visual system in humans is not trivial, as early visual neural responses are often highly localised and involve intricate interactions with subcortical structures (e.g., LGN) and higher‐order areas (e.g., MT+). Traditional neuroimaging techniques have been used to explain the phenomenon, but they proved to be suboptimal. Non‐invasive electrical recording modalities have limited spatial resolution (e.g., EEG) or are not widely available (e.g., MEG, OPM (Boto et al., 2017)); and indirect or hemodynamic measures (e.g., fMRI, fNIRS, PET) lack the temporal resolution to study the fast‐changing dynamics and probe causal relationships. Invasive electrophysiological recording modalities, such as electrocorticography (ECoG) or stereotactic EEG, most commonly implanted during the treatment of intractable epilepsy, provide a unique means to study the human brain, even though the coverage of the primary visual cortex is rarely considered as occipital epilepsies are relatively uncommon (Taylor, Scheffer, & Berkovic, 2003) and not targeted for ressective surgery (eloquent cortex) (Heo, Kim, Chung, & Lee, 2017).

In an early study (Kamp, Sem‐Jacobsen, Leeuwen, & T‐Weel, 1960), sinusoidally modulated light was presented to a human subject with an implant of multiple depth electrodes. Even though a series of contacts extended into the occipital cortex, the electrophysiological activations in this area were not reported and mainly qualitative results were discussed. A more recent study (Krolak‐Salmon et al., 2003) reported the presence of steady‐state responses following the screen's refresh rate (60–70 Hz) in LGN and the optic radiation of one patient and at highly localised areas along the calcarine fissure in two other patients. As this study was not aimed at flicker stimulation, these results were rather unexpected and potential neural mechanisms involved in the fundamental response were not discussed. Similarly, other invasive studies using flickering stimuli in humans did not discuss potential mechanisms, but rather exploited the steady‐state response as a tool for probing other neural functions (Jonas et al., 2016; Winawer et al., 2013).

Given the growing amount of scholarly literature reporting on results obtained from flickering visual stimuli, it would be highly beneficial, both for clinical studies and basic visual research, to gain a deeper understanding of the neural mechanisms involved in the genesis of the fundamental response.

In this study, we report on a case of one epilepsy patient who was implanted with a large subdural grid covering the entire occipital cortex of the right hemisphere, including the inter‐hemispheric fissure. The patient participated in an experiment during which the luminosity of rectangles presented in the foveal and peripheral visual field was sinusoidally modulated at a temporal frequency between 11 and 15 Hz. In addition to the expected phase‐locked fundamental response, we observed highly synchronised activity in the high‐gamma band which was strong related to the fundamental response on a single‐trial level.

**FIGURE 1:**
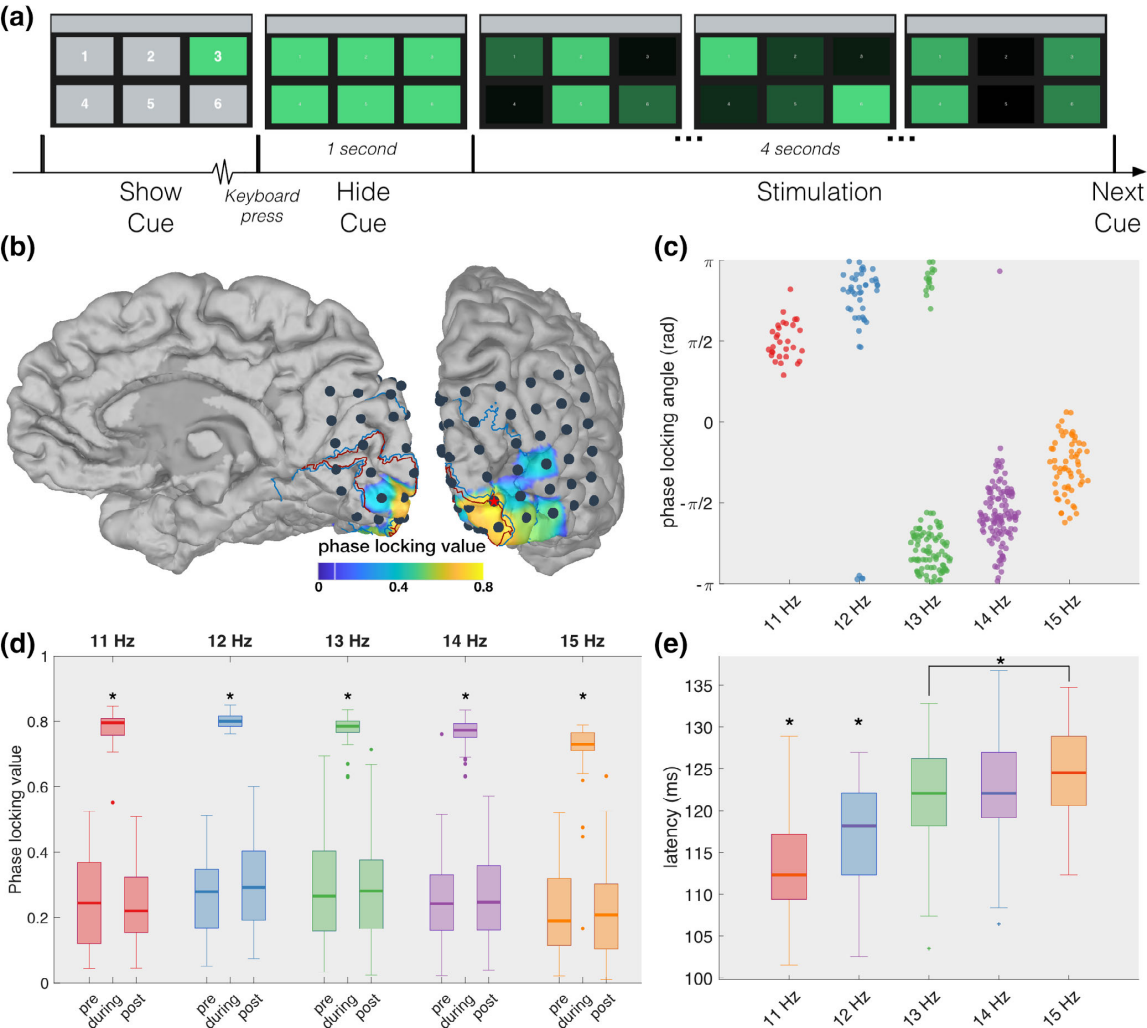
Phase locking to stimulation. (a) Graphical rendition of the visual stimulation presented during one trial. Note that the target numbers in the left‐most panel are enlarged for better visualisation and do not correspond to their actual size. (b) Average phase locking value (PLV) of the fundamental response during the stimulation for each subdural channel. High PLVs are localised to the posterior part of the primary visual cortex. To declutter the plot, only the cortical areas that exhibit a significant increase in PLV compared to pre‐ and post‐stimulation are indicated with a colour. The red and blue outlines on the cortex indicate V1 and V2, respectively, while the black dots mark the 48 recording sites. The electrode indicated with a red star exhibits the strongest average phase locking. (c) For the starred electrode, the phase locking angles of individual trials, indicated by individual dots, are highly similar and show an upward trend for increasing frequency. (d) Boxplots showing that the starred electrode synchronises to the stimulation for every frequency and returns to baseline when the stimulation ends. (e) The latency of the fundamental component shows an increasing trend for increasing frequency. Stars indicate significant differences

## 2 RESULTS

One patient with intractable epilepsy participated in an experiment with six flickering rectangles arranged in a two‐by‐three design (Figure 1a). The rectangles spanned a visual angle of 8.4^∘^ horizontally and 5.5^∘^ vertically, with a horizontal and vertical spacing between rectangles of 2.9 and 1.4^∘^, respectively. During each trial, the patient fixated his gaze on one of the rectangles which were all flickering for 4 seconds. The flicker was achieved by adjusting the luminosity of the rectangles based on a sampled sinusoidal profile (Manyakov et al., 2013). Then, 4 sessions of 60 trials were performed in each of which the rectangles were assigned a unique combination of a frequency between 11 and 15 Hz and a phase of 0, 120, 180, or 240° (Table 1). However, as the patient was bedridden, the experiment was performed in a hospital room using a laptop monitor with a non‐linear gamma profile. Prior to the analysis of the data, the “sinusoidal” stimulation profile was therefore corrected to reflect the actual stimulation profile presented to the subject (Supplementary Figure S1).

**FIGURE 2:**
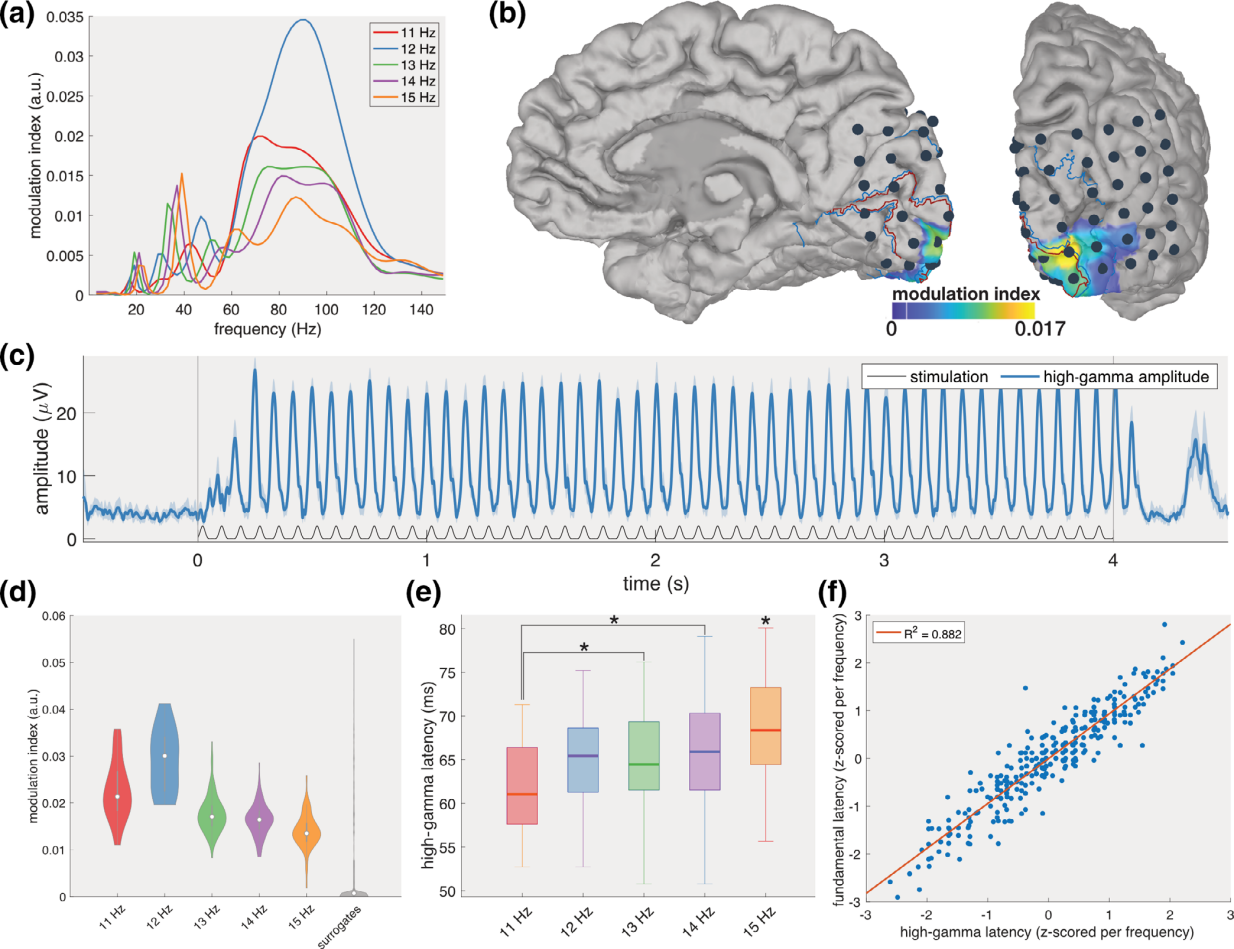
High‐gamma amplitude. (a) Modulograms showing the modulation index (i.e., coupling strength) between the phase of the gazed stimulation and the amplitude of the neural response at frequencies below 150 Hz at electrode 36. (b) Spatial distribution of the modulation index between the phase of the stimulation and the amplitude of the high‐gamma response. (c) Averaged high‐gamma amplitude in the temporal domain when gazing at the 12 Hz zero‐phase target. The full blue line indicates the amplitude in the high‐gamma band and the surrounding shaded areas the 95% confidence interval. The thin black line indicates the gazed stimulation profile. (d) The modulation index for each of the five frequencies adopted in this study and of a surrogate group. (e) The latency of the high‐gamma response exhibits a positive trend towards higher temporal frequencies. Stars indicate significant differences. (f) Linear regression between the high‐gamma latency and the fundamental latency reveals a strong relationship between both latencies

**TABLE 1:** Frequency‐phase combinations for each of the six rectangles, represented as (frequency [Hz]/phase [radians])

	Target					
Session	1	2	3	4	5	6
1	12/0	$14/\frac{2\pi }{3}$	$12/\frac{4\pi }{3}$	$14/\frac{4\pi }{3}$	$12/\frac{2\pi }{3}$	14/0
2	13/0	$14/\frac{2\pi }{3}$	$13/\frac{4\pi }{3}$	$14/\frac{4\pi }{3}$	$13/\frac{2\pi }{3}$	14/0
3	11/0	15/*π*	13/0	13/*π*	11/*π*	15/0
4	13/0	15/*π*	14/0	14/*π*	13/*π*	15/0

### 2.1 V1 is highly phase locked to the gazed stimulation

In this study, we focus on the neural activation generated in response to the foveal stimulation. A phase locking analysis between the gazed stimulation profile and the neural activations at the fundamental frequency (filtered signal between 10 and 16 Hz, Supplementary Figure S2) shows a total of nine electrodes that exhibit a significant increase in phase locking value (PLV) that is consistent across all used frequencies. These electrodes span V1, V2, and V4 with the activity in the primary visual cortex exhibiting, as expected (Wittevrongel, Khachatryan, Fahimi Hnazaee, Carrette, et al., 2018), the most stable locking to the stimulation (electrode 36, indicated with a red star in Figure 1b).

At this location in V1, the fundamental response is highly synchronised to the gazed flickering (median PLV = 0.78 ± 0.03) and is significantly different from the PLV during the pre‐ and post‐stimulation intervals (median PLV = 0.24 ± 0.03 and 0.25 ± 0.03 respectively; all *Z* ≥ 4.782, *p* < .0001, Bonferroni‐corrected Wilcoxon signed‐rank test, Figure 1d, Supplementary Figure S3 for the eight other significant channels). The phase locking angle between the fundamental response and the stimulation exhibits a strong positive circular‐linear correlation with respect to the gazed frequency (*ρ* = .849) (Figure 1c). A circular Watson–Williams (i.e., a circular analysis of variance [ANOVA]) test shows a significant effect of the gazed frequency on the phase locking angle (*F*(4,359) = 328.12, *p* < .0001). While previous works have used the slope of the circular‐linear relationship to estimate the latency of the fundamental response (di Russo & Spinelli, 1999; di Russo, Teder‐Sälejärvi, & Hillyard, 2003), this work opted to calculate the latency in the time domain as this allowed us to obtain the latency of individual trials instead of a single measurement across groups. The median latency for each gazed frequency ranges from 112 ms when gazing the 11 Hz frequency to 125 ms for the 15 Hz frequency, with a positive trend towards higher frequencies (Figure 1e, Supplementary Figure S4). A Kruskal–Wallis test reveals a significant effect of the gazed frequency on the fundamental latency (*χ*^2^(4,359) = 70.83, *p* < .0001), and further pairwise comparison using the Bonferroni‐corrected Wilcoxon rank‐sum test shows a significant difference between the latencies for 11 Hz targets with all others (*Z* = 3.301, *p* = .0096 for 12 Hz; *Z* ≥ 5.853, *p* < .0001 for 13 till 15 Hz), for the latencies of 12 Hz targets with the all others (*Z* = 3.473, *p* = .0051 for 13 Hz; *Z* ≥ 4.377, *p* < .0001 for 14 and 15 Hz) and between the latencies of 13 and 15 Hz targets (*Z* = 2.821, *p* = .0492). The variability in fundamental latency is similar across all frequencies with the inter‐quartile ranges varying between 7.81 and 8.79 ms.

### 2.2 High‐gamma amplitude oscillations at the gazed frequency

To identify other potential frequency components that synchronise to the stimulation, a phase‐amplitude coupling analysis between the phase of the stimulation and the amplitude of the neural response over the frequency spectrum was performed. The modulograms reveal consistent strong coupling in the higher frequency components between 55 and 125 Hz (Figure 2a). Based on these results, the remainder of this work defines the high‐gamma band as the spectral range from 55 until 125 Hz.

**FIGURE 3:**
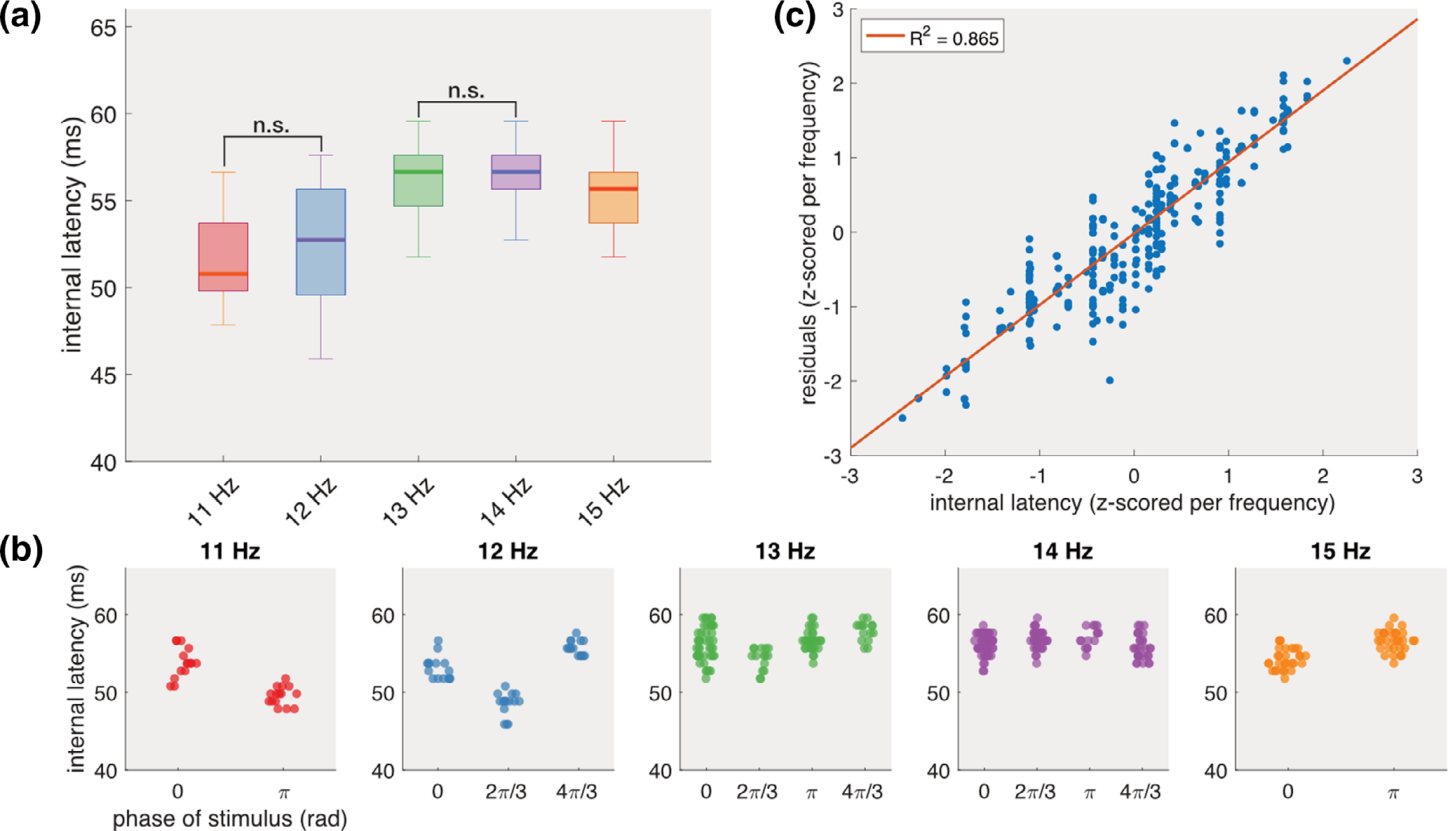
Internal latency. (a) The latency between the peaks of the high‐gamma amplitude and fundamental response. (b) When grouped per unique frequency‐phase combination, distinct clusters are revealed for the 11 and 12 Hz frequencies. Each dot indicates one trial. (c) Regression between the internal latencies and the residuals from Figure 2f. Each dot indicates one trial

Inspection of the average high‐gamma amplitude across all trials reveals increased activity as early as 40 ms after the onset of the stimulus, evidenced by a significant difference compared to the amplitudes at the stimulation onset (*Z* > 5.29, *p* < .001, Bonferroni‐corrected Wilcoxon signed‐rank test, Supplementary Figure S5). The average high‐gamma amplitude per unique frequency‐phase combination additionally shows oscillations at the gazed frequency that begin shortly after the stimulation onset and cease following stimulation offset (Figure 2c for 12 Hz and zero phase, Supplementary Figure S6 for the other frequencies). A single‐trial phase‐amplitude coupling analysis corroborates this finding and reveals that the coupling between the phase of the gazed stimulation and the high‐gamma amplitude is highly localised in V1 (electrode 36, Figure 2b). For all gazed frequencies, the modulation index (MI; i.e., a measure of the coupling strength) at this location is significantly higher than those of a surrogate group in which the gamma amplitude and the gazed stimulation profile of random trials were paired with each other (Kruskal–Wallis *χ*^2^(5, 2359) = 420.48, *p* < .001; all *Z* > 7.01, *p* < .001, Bonferroni‐corrected Wilcoxon rank‐sum test, Figure 2d, Supplementary Figure S7). The strongest coupling is elicited by the 12 Hz stimulus. Its MI is significantly larger than that of the other stimulation frequencies (all *Z* > 8.57, *p* < .001, pairwise Wilcoxon rank‐sum tests with Bonferroni correction).

Similar to the latency of the fundamental response, the latency of the high‐gamma response for each trial was measured in the time‐domain as the time between the peak of the stimulus and the peak of the gamma amplitude (Supplementary Figure S8). The high‐gamma latency exhibits a consistent increase with gazed frequency, from 61.04 ms for the 11 Hz to 68.36 ms for the 15 Hz stimulation (Figure 2e). All latencies were normally distributed (Lilliefors test *p* > .01), and ANOVA shows a significant effect of the gazed frequency on the high‐gamma latency (*F*(4,359) = 8.91, *p* < .001). Further pairwise comparison, using Bonferroni‐corrected unpaired *t* tests, reveals a significant difference between the latencies when gazing at a 15 Hz target and all other frequency targets (all *t* > 2.85, *p* < .05), and at an 11 Hz target compared to 13 and 14 Hz targets (both *t* > 3.17, *p* < .05).

The trial‐to‐trial variability of the high‐gamma latencies is similar to that of the fundamental latencies (interquartile ranges between 7.32 and 8.79 ms). A linear regression model shows a strong positive correlation between both single‐trial latencies (*R*^2^ = .882, *p* < .001, Figure 2f) with a shorter high‐gamma latency consistently resulting in a shorter fundamental latency and vice versa.

### 2.3 From high‐gamma to fundamental response

While the high‐gamma and fundamental oscillations show strong synchronisation, the latency of the latter is considerably larger, suggesting the involvement of an additional internal neural process in the generation of the fundamental response. The single‐trial internal latencies, defined in the time‐domain as the time‐interval between the peaks of the gamma amplitude and the fundamental response, vary between 50.78 and 56.64 ms (Figure 3a, Supplementary Figure S10). Compared to the latencies reported above, the internal latencies exhibit a considerably smaller trial‐to‐trial variability with interquartile ranges varying from 1.98 to 6.10 ms when gazing at 15 and 12 Hz, respectively. The internal latencies for each gazed frequency are not consistently normally distributed (Lilliefors test, *p* < .001). The Kruskal–Wallis test reveals a significant effect of the gazed frequency on internal latency (*χ*^2^(4,359) = 149.31, *p* < .0001). Further pairwise comparisons, using Bonferroni‐corrected Wilcoxon rank‐sum tests, show significant latency differences between all combinations except between 11 and 12 Hz (*Z* = 1.17, *p* > .05) and 13 and 14 Hz (*Z* = 0.22, *p* > .05). Surprisingly, when taking into account the phase of the gazed stimulus, the internal latencies for the lower frequencies (i.e., 11 and 12 Hz) exhibit clusters around different stimulus phases, which is less evident for the other frequencies used in this study (Figure 3b).

**FIGURE 4:**
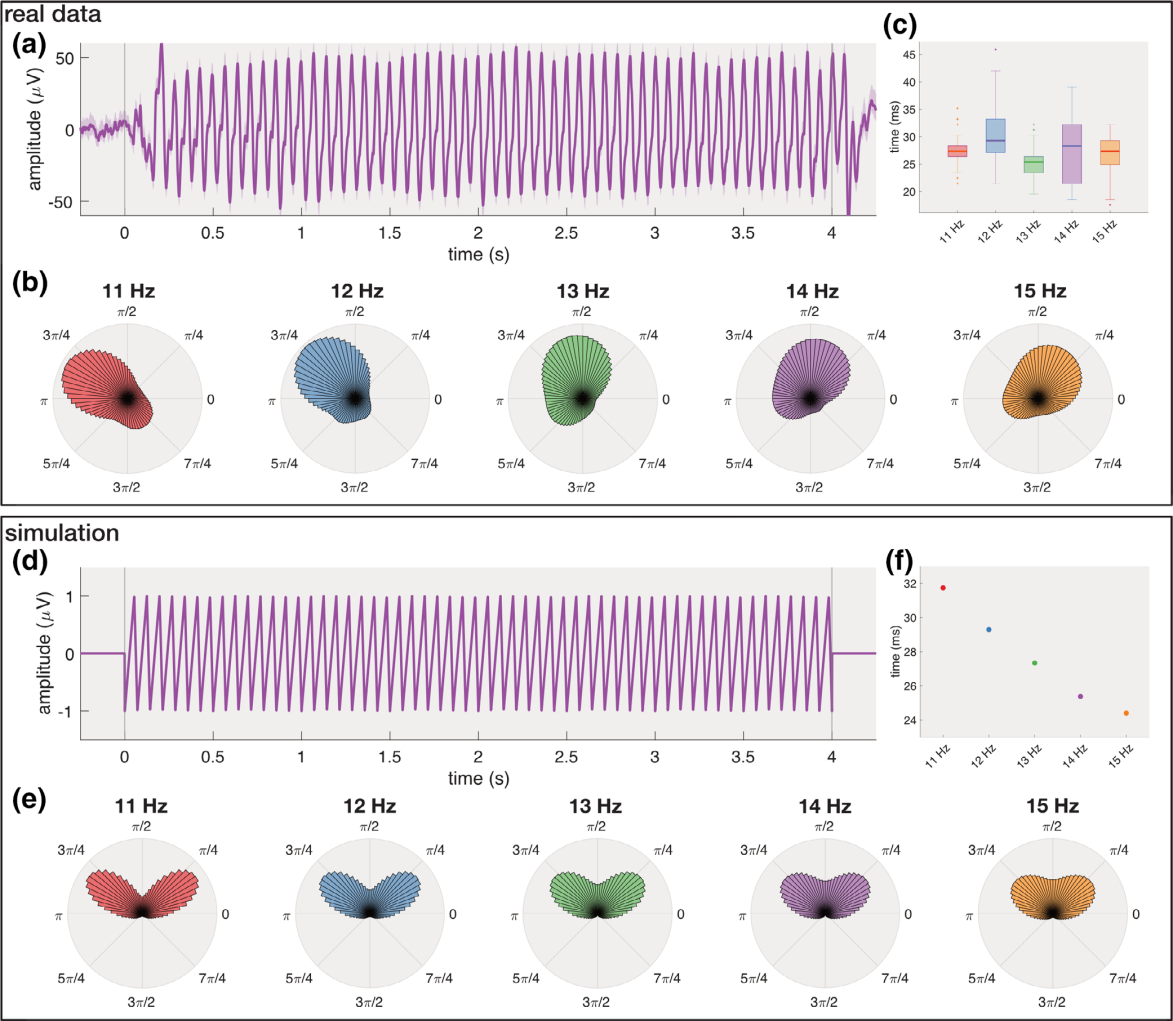
Simulation. Phase‐amplitude coupling (PAC) analysis with neural responses at subdural channel 36 (upper panel) and simulated triangular signals at the gazed frequency (lower panel). (a) Average broadband (4–250 Hz) signal when gazing a 14 Hz stimulus shows a non‐sinusoidal response with sharp transitions. (b) Phase‐amplitude coupling between the phase of the fundamental response (10–16 Hz) and the amplitude of the gamma component (55–125 Hz). Each circular plot shows the average amplitude of the high‐gamma response (indicated by the length of the bars) with respect to the phase of the fundamental component (indicated by the radial angle). Note that the five stimulation frequencies achieve their maximal gamma amplitudes at different phase angles. (c) Time between the minimal and maximal gamma amplitude is largely independent of the gazed frequency. (d) Example of the artificial sawtooth signal used in the simulation. Similar to the real data, the sawtooth signal has sharp transitions from ascending to descending amplitudes. (e) The spurious phase‐amplitude coupling exhibits maximal amplitude at equal phases for all five stimulation frequencies. (f) Time between the minimal and maximal gamma amplitude is strongly correlated with the stimulation frequency. Note that only one point is shown per stimulation frequency since identical results were obtained for each trial at that frequency

Linear regression models between the internal latencies and fundamental or high‐gamma latencies do not show a relevant relationship, despite the significance of the former (Supplementary Figure S11, *R*^2^ = .046, *p* < .001 and *R*^2^ = .010, *p* = .06, respectively). However, the internal latencies do positively correlate with the residuals of the regression model between the high‐gamma and the fundamental latencies (Figure 3c, *R*^2^ = .865, *p* < .001). A trial for which the initial model exhibits a positive residual is thus linked to a larger internal latency, and vice versa. Despite the small inter‐trial variability, the internal latency indirectly affects the latency of the fundamental component, which might also explain the significant but low correlation between the single‐trial internal latencies and fundamental latencies. A multivariate regression model that includes both the high‐gamma and internal latencies indeed achieves a higher explanatory power (*R*^2^ = .972, *p* < .001) for the fundamental latency than a univariate model solely based on the gamma latency.

## 3 DISCUSSION

Despite its widespread adoption in psychophysics, visual neuroscience and clinical research, the neural mechanisms behind the steady‐state response elicited by flickering visual stimulation are still unclear. In this study, we took advantage of the high spatial, temporal and spectral resolution of subdurally recorded signals to advance our understanding of the neural effects of this widely adopted paradigm. We corroborate previous (mostly non‐invasive) findings that the primary visual cortex is most strongly involved in generating a neural response at the gazed frequency (i.e., the fundamental response). However, in addition to the fundamental oscillation, we also showed that the amplitude in the high‐gamma band (defined from 55 to 125 Hz in a data‐driven manner) resonates at the gazed frequency. The latencies of both neural oscillations are frequency‐dependant, with a higher temporal frequency resulting in larger latencies. The latency of the high‐gamma oscillation varies between 60 and 68 ms. The fundamental response exhibits latencies between 112 and 125 ms compared to the stimulation onset. The trial‐to‐trial variabilities of both latencies are similar (approximately 7 ms) with both measures being highly and positively correlated, albeit with a consistent delay of approximately 55 ms.

### 3.1 Fundamental response as combination of bottom‐up visual input and top‐down feedback

The photons that interact with the retina are translated into electrical pulses by specialised retinal structures which then get delivered to the LGN, a substructure of the thalamus. It is believed that the LGN mostly acts as a relay (Guillery & Sherman, 2002) that projects the retinal information to selective laminar layers in the primary visual cortex (V1) where the retinotopic organisation is preserved (Tootell, Silverman, Switkes, & de Valois, 1982). Primary sensory areas, such as V1, actively process the sensory inputs, which is reflected by increased activity in the higher spectral range (Buzsáki, Anastassiou, & Koch, 2012; Ray & Maunsell, 2011). Our phase‐amplitude coupling analysis showed indeed considerable synchronisation between the visual stimulation and the high‐gamma band amplitude in V1 with an estimated latency between 60 and 68 ms. In humans, few measurements are available of the latency between the retinal input and the associated neural activity in V1. Using averaged visual evoked potentials in a broad spectral band (0.1–70 Hz) in response to a slowly reversing checkerboard (reversal rate 1.1 Hz) and transient face stimuli (400 ms), the visual latency in humans was previously estimated to be around 62 ms in V1 (Krolak‐Salmon et al., 2003) (and at around 42 ms in LGN). More recent studies confirmed this by reporting latencies in the human primary visual cortex of 66 ms during a rapid serial visual presentation paradigm (Yoshor, Bosking, Ghose, & Maunsell, 2006), of 64 ms during the presentation of a flashing checkerboard (Self et al., 2016) and of 64 ms during a spatial attention task with visual stimuli (Martin et al., 2019). Our measurement, based on high‐gamma responses, thus coincides with previous visual latency estimates.

Interestingly, while both the high frequency and fundamental neural activations synchronise to the visual stimulation, the measured latencies suggest an indirect relationship between both. Note that the latency of the latter is measured between 112 and 125 ms, which is in line with estimations from previous studies (di Russo et al., 2003; di Russo & Spinelli, 1999). The temporal delay of approximately 55 ms compared to the high‐gamma response most likely originates from the involvement of other cortical structures via feedback projections. This theory is corroborated by reports in literature that attentional modulation affects the neural responses of the visual cortex (Jack, Shulman, Snyder, McAvoy, & Corbetta, 2006; Kim et al., 2007; Sirotin & Das, 2009). The exact nature of these feedback projections is still unclear. Potential mechanisms include the thalamic structures through corticogeniculate pathways and lateral connections to higher‐order visual areas (V2, V4). For the former, conduction latencies in an alert macaque monkey vary depending on the type of pathway and range from fast (<7ms) to slow (>14 ms) (Briggs & Usrey, 2009). Based on previous reports of highly similar visual latencies in macaque and human V1 (Schmolesky et al., 1998), these corticogeniculate latencies are likely to be similar in human subjects and do not conform to the internal latencies obtained in this study, even when assuming reciprocal connections. Furthermore, the discrepancy between the trial‐to‐trial variabilities of the high‐gamma and internal latencies additionally suggests different origins for both. The fast high‐gamma responses most likely originate from the processing of geniculate input to V1. Since the activity in LGN is affected by attention (O'Connor, Fukui, Pinsk, & Kastner, 2002), it might partially explain the variability of the high‐gamma latencies. However, the concurrent internal latencies are considerably more stable, suggesting the involvement of another neural structure. Given the mismatch between the internal and expected corticogeniculate conduction latencies and the fact that the majority of the incoming connections to V1 are from a non‐geniculate origin (Muckli & Petro, 2013), feedback projections from extrastriate visual areas seem a more plausible candidate.

The primary visual cortex can be considered the first synaptic level for visual processing (Mesulam, 1998). V1 is linked to extrastriate areas such as V2 and V4, which further process the input from V1 and additionally provide feedback to the previous synaptic level (Mesulam, 1998; Muckli & Petro, 2013). As the estimated latency per synaptic level is roughly expected to induce a delay between 10 and 15 ms (Martin et al., 2019), the measured internal latencies of approximately 55 ms would be explained by propagation and feedback over two synaptic levels. Additional evidence that the processing of flickering visual input involves several visual synaptic levels is given by the subjects' unprompted reports of perceiving illusory shapes, colours or motion while gazing at flickering stimuli, even though this (often unwanted) side‐effect is very rarely mentioned in the literature (e.g., (Blair, Erlikhman, & Caplovitz, 2019; Herrmann, 2001)). Given that higher‐order areas are involved in the processing of colour (lingual and fusiform gyri (Chao & Martin, 1999; Lueck et al., 1989), primate‐analogue V4), shapes (fusiform gyrus (Kanwisher, Woods, Iacoboni, & Mazziotta, 1997; Slotnick & White, 2013), primate‐analogue PIT) and motion (lateral occipitotemporal area (Watson et al., 1993), primate‐analogue V5), it is likely that simple visual luminosity changes indeed cross at least two synaptic levels to higher‐order visual areas which project back and eventually modulate neural activity in V1. Furthermore, this theory agrees with recent work that has provided evidence that feedforward connections rely on activations in the higher spectral range (i.e., gamma band), while feedback mainly operates in the lower spectral range (i.e., alpha/beta) (Michalareas et al., 2016; van Kerkoerle et al., 2014).

### 3.2 Temporal stimulus characteristics influence the neural responses

Neural response latencies are not independent of the stimuli being presented. Sinusoidal gratings with increasing spatial frequencies consistently elicit larger latencies of non‐invasively recorded visual evoked potentials in human subjects (Jones & Keck, 1978; Parker & Salzen, 1977; Vassilev, Mihaylova, & Bonnet, 2002). Primate research has furthermore shown that individual neurons along the retino‐geniculo‐cortical pathway exhibit temporal frequency tuning characteristics (e.g., in the retinal ganglion (Enroth‐Cugell, Robson, Schweitzer‐Tong, & Watson, 1983), LGN (Derrington & Lennie, 1984), and V1 cells (Hawken, Shapley, & Grosof, 1996)) which are often intrinsically linked with their respective spatial frequency tuning characteristics (Tan & Yao, 2009). While individual neurons exhibit preferences to some temporal frequencies, the subdural recordings in this study reflect local field potentials of larger cortical populations, rendering a direct comparison between the above‐mentioned studies and the current results not trivial, even though high‐gamma responses strongly correlate with neuronal spiking activity (Ray, Crone, Niebur, Franaszczuk, & Hsiao, 2008).

Nevertheless, in the current study, we show that the effect of the temporal frequency of the stimulus is not restricted to single neurons, but also affects the response latencies of larger cortical populations. More specifically, the latency of the high‐gamma response increases with increasing frequency (at least within the frequency range adopted in this study). While this trend resembles a linear function, the narrow spectral range of the tested frequencies in this study (i.e., from 11 to 15 Hz) does not warrant a more generalised conclusion. Similar to single‐cell temporal tuning characteristics in primate V1 (Hawken et al., 1996), the linear trend most likely does not hold for higher temporal frequencies.

As the high‐gamma response most likely reflects geniculo‐cortical projections, the question arises as to whether gazing different temporal frequencies also induces latency differences in LGN. The striking similarities in receptive field properties of retinal ganglion and LGN cells originally led researchers to believe that LGN does not actively contribute to the processing of visual input. However, more recent work has shown that LGN cells indeed transform the retinal information in both spatial and temporal domains (Usrey & Alitto, 2015).

### 3.3 Implications

Thanks to its high signal‐to‐noise ratio and stable inter‐subject response, the SSVEP has been adopted in numerous clinical studies (Vialatte et al., 2010). The brain's response to visual flickering has been used as a tool for gaining insight into neurodegenerative disorders, such as Alzheimer's (Kikuchi et al., 2002) and Parkinson's (Vanegas et al., 2019), as well as other neurological and psychiatric conditions including, but not limited to depression (Moratti, Rubio, Campo, Keil, & Ortiz, 2008), migraine (de Tommaso et al., 2014), autism (Dickinson, Gomez, Jones, Zemon, & Milne, 2018), and schizophrenia (Javitt, 2009).

The majority of these studies primarily focus on differences in the amplitude and/or latency of the fundamental response between the patients and healthy individuals. However, to date, the precise neural mechanisms that give rise to the fundamental response are not well understood. The evidence presented in the current study suggests that two processes (i.e., bottom‐up visual processing and top‐down feedback) are involved in generating the fundamental response. Thus, inter‐group variations in amplitude and/or latency observed in the above‐mentioned studies could originate either from altered properties of the retino‐geniculo‐cortical pathways, which would result in deviations of the early high‐gamma responses (which indirectly influence the fundamental response), or from pathological effects on the feedback processes that bring about the fundamental response.

One potential approach to discriminate between the initial sensory response and the feedback process would be to complement the flicker paradigm (>4Hz) with a transient stimulation paradigm (e.g., ≤1Hz). The latter allows one to obtain an isolated latency measurement of the early visual response, as the fundamental response would be absent. In combination with the properties of the fundamental response, more firm conclusions could be drawn.

### 3.4 Potential spurious cross‐frequency coupling

It is widely known that spurious (i.e., artefactual) cross‐frequency coupling can occur with non‐sinusoidal or asymmetric (i.e., non‐linear) signals (Aru et al., 2015; Jensen, Spaak, & Park, 2016; Scheffer‐Teixeira & Tort, 2016). The frequency spectrum of these signals typically also shows pronounced harmonic responses which can extend into the gamma range. When assessing the coupling between a lower and higher frequency component (e.g., theta–gamma coupling (Belluscio, Mizuseki, Schmidt, Kempter, & Buzsáki, 2012)), a strong relationship can be found due to the applied filtering procedure, even when there are no underlying oscillators (e.g., in white noise) or when the oscillators are not coupled (Scheffer‐Teixeira & Tort, 2016). It is important to note that the raw neural responses to the flickering stimulation used in our study are strongly oscillatory but not purely sinusoidal. Averaging the broadband responses reveal triangular or sawtooth waveforms with sharp transitions from low to high amplitude (Figure 4a). As such, the corresponding frequency spectrum indeed shows strong harmonic components which extend beyond the traditional high‐gamma frequency range (>120 Hz) (Supplementary Figure S2). However, in addition to these harmonics, the spectrum also shows overall increased amplitudes in the gamma range extending until 250 Hz, indicating that the raw signal does contain a more general gamma signature.

The cross‐frequency analysis between the phase of the fundamental response (10–16 Hz) and the amplitude of the high‐gamma component (55–125 Hz) shows strong coupling, and it is therefore important to corroborate this finding with additional evidence that the coupling is not spurious. A first piece of evidence for true coupling is given by the modulogram in Figure 2a, which shows that the coupling between the phase of the stimulation profile and the amplitude of the neural response at subdural channel 36 is covering a broad gamma band range. For example, the modulogram for the 12 Hz stimulus shows a smooth curve displaying increased coupling between 55 and 125 Hz. Each point in this curve is obtained by filtering the neural response in a 5‐Hz bandwidth centred at the corresponding frequency *f* (i.e., filtered between *f* − 2.5 and *f* + 2.5 Hz). Note that for a large amount of centre frequencies the band pass does not include any 12 Hz harmonics. Notably, the maximal coupling is obtained when the signal is filtered between 88.5 and 93.5 Hz (i.e., centre frequency of 91 Hz). If the coupling would mainly originate from the harmonics, the modulogram would have contained peaks at multiples of the stimulation frequency under consideration.

A second source of evidence for true coupling comes from the fundamental phase angles at which the high‐gamma amplitude is maximal. A coupling analysis between the phase of the fundamental component and the amplitude of the high‐gamma component at subdural channel 36 reveals that the phase angles at which the high‐gamma amplitude are significantly different across the five stimulation frequencies (*F*(4,359) = 626.5, *p* < .0001, Watson–Williams test), and reveal a clear negative trend (Figure 4b, Supplementary Figure S9). If the gamma oscillations were due to the non‐sinusoidal shape (and its harmonics), one would expect a fixed relationship between the fundamental and gamma components. To illustrate this, we ran a simulation with a simulated sawtooth signal at frequencies between 11 and 15 Hz. In this case, the signal is not a result of coupled oscillators and any gamma components are artefacts due to the filtering of the non‐sinusoidal signature. Also here, a strong phase‐amplitude coupling is found between the fundamental and high‐gamma component, but the maximal high‐gamma amplitudes are consistently synchronised to the same phase angles (Figure 4e), as expected (Kramer, Tort, & Kopell, 2008). This discrepancy between the results of the simulated and neural data gives further confidence that the reported coupling is not artefactual.

Finally, we also took a closer look at the characteristics of the gamma oscillations of the neural and simulated data. Here, we found that in the neural data, the time during which the gamma amplitude increases is similar for all stimulation frequencies (Figure 4c), while there was a clear negative trend for the simulated data (Figure 4f). This shows that the gamma response does not originate from a filtering artefact and that the equal length of the gamma “bursts” is likely intrinsic to the underlying neuronal population.

### 3.5 Open questions

While reports have shown contiguous steady‐state responses until 100 Hz (Herrmann, 2001), it has been shown that the spectrum can be divided into three bands based on the signal‐to‐noise ratio: low (<15 Hz), medium (15–25 Hz), and high (25–60 Hz) (Regan, 1989; Wang, Wang, Gao, Hong, & Gao, 2006). This has led to the hypothesis that these bands differ in the neural mechanism behind their steady‐state responses (Vialatte et al., 2010). The stimulation frequencies used in this study were limited to a narrow frequency band in the lower spectral range, and we are currently not able to comment on whether the same neural mechanisms are involved in generating of the steady‐state response for flickering of a higher frequency band. Future studies using data acquisition and analysis techniques described in the current study could shed light on this question.

It is known that the latency of the fundamental steady‐state response is subject to attentional modulation (di Russo et al., 2003; di Russo & Spinelli, 1999). Given the results presented in this work, the question arises whether attentional modulation would resort to a similar effect on the gamma oscillations. An experimental paradigm in which the subject's attention level is manipulated could shed light on whether the gamma component solely represents visual processing or whether top‐down effects also have an effect. In addition, it could enable one to analyse the exact relationship between the gamma oscillation and the ensuing fundamental response.

### 3.6 Strengths and limitations

Compared to previous studies, the present study takes advantage of more advanced signal analysis methods in combination with a direct intracranial occipital surface recording in a human subject, thus providing a high spatial, temporal and spectral resolution unattainable with traditional scalp‐recorded EEG or fMRI. As intracranial recordings are only warranted for clinical purposes (e.g., refractory epilepsy) and that occipital lobe epilepsies are rare (Taylor et al., 2003) or often considered inoperable (Heo et al., 2017), considerably fewer opportunities arise to record from subdural electrode implantations over primary visual areas in humans. The present study is therefore based on the data of a single subject and might raise questions about the generalisation of the presented results. Even though this is a valid concern, it is worth noting that the process under consideration is a primal visual response, which is, unlike cognitive responses, less likely to vary across individuals and is even homologue across species (Orban, van Essen, & Vanduffel, 2004; van Essen, 2003). Furthermore, both the high‐gamma and fundamental latencies reported in this work are in accordance with scientific literature, even though they were never described jointly in a single study.

While the current study focuses on neural responses to the foveal stimulation, simultaneous peripheral flickering stimulation was presented during the experiment. However, the retinotopic organisation of the primary visual cortex allows us to avoid the cortical areas that actively process the peripheral stimulus. Indeed, in a previous paper (Wittevrongel, Khachatryan, Fahimi Hnazaee, Carrette, et al., 2018), we have already highlighted the peripheral processing in distinct cortical areas in V1 which were not included in the analyses of the present study.

## 4 CONCLUSION

The steady‐state response is a cortical activation elicited by flickering visual stimulation and is widely used to investigate cerebral processes and to assess neural pathologies. However, the neural mechanisms behind its generation are currently not well understood. Here we show that the fundamental steady‐state response of frequencies between 11 and 15 Hz is preceded by coupled oscillations in the gamma band (55–125 Hz). The fundamental component consistently trails the gamma oscillation with about 55 ms and the latencies of both components are strongly correlated on a single‐trial level. These results imply that variations in the steady‐state responses due to the pathology or experimental task could originate from two sources. Identifying the precise origin of the variation will ultimately lead to a deeper understanding of the investigated paradigm.

## 5 MATERIALS AND METHODS

### 5.1 Subject

A 38‐year old male patient (right‐handed, corrected‐to‐normal vision) with refractory non‐photosensitive epilepsy participated in the study. He was admitted to the hospital (UZ Gent) for the monitoring of seizure activity and the functional mapping of the eloquent cortex (visual, language, etc.). A large subdural grid containing 48 (6 × 8) platinum electrodes embedded in silastic (Ad‐Tech) was implanted on the right occipital cortex: convexity and mesial inter‐hemispheric cortex. Each electrode had a 4.0 mm diameter with 2.3 mm exposure and 10 mm contact spacing. Functional mapping confirmed that a large part of the grid was located over the visual cortex. ECoG data were continuously recorded throughout the experiment at a sampling rate of 1,024 Hz using an SD LTM 64 Express (Micromed, Italy) medically certified device. Prior to analysis, an epileptologist (co‐author A. M.) manually verified the data to identify the subdural contacts located on the pathological tissue. While two such electrodes were identified, none of them were located on the cortical area relevant for the present purpose.

The study was conducted in accordance with the current version of the Declaration of Helsinki (2013) following prior ethical approval from the ethical committee of Gent University Hospital. Prior to participating, the patient was informed about the aim of our study, the experimental procedure and what would be done with the recorded data to which he gave his written consent.

### 5.2 Localisation of ECoG electrodes

Based on the pre‐implantation MRI scan of the patient, cortical reconstruction and volumetric segmentation was performed with the FreeSurfer image analysis suite (version 5.3.0) (Fischl, 2012). The FreeSurfer output was then loaded into Brainstorm (Tadel, Baillet, Mosher, Pantazis, & Leahy, 2011) and co‐registered with a post‐implant CT using the SPM12 (Penny, Friston, Ashburner, Kiebel, & Nichols, 2011) extension. The coordinates of the implanted electrodes were manually obtained from the artefacts in the CT scan and projected on the cortical surface. All cortical visualisations were done using the Brainstorm toolbox (Tadel, Baillet, Mosher, Pantazis, & Leahy, 2011).

### 5.3 Experimental design

The experimental interface consisted of six identical rectangular targets (8.8 × 5.8 cm) presented on a 60 Hz laptop monitor (Dell Latitude E6430). A number (from 1 to 6) was displayed in the centre of each target which served as the fixation point for the corresponding rectangle. The bed‐ridden subject viewed the laptop monitor from approximately 60 cm. At this distance, the visual angle of by the rectangles was 8.4 × 5.5^∘^ and the distance from the fixation point to the edge of the neighbouring target 5.5^∘^ horizontally and 4.1^∘^ vertically.

The experiment consisted of four sessions in each of which the six rectangles were assigned a unique combination of frequency and phase (Table 1). Each session consisted of 90 trials. At the beginning of each trial, one of the targets was cued by maintaining its green colour while the other rectangles were shown in grey (Figure 1a). The subject was instructed to direct his/her gaze at the cued target and maintain fixation for the entire duration of the subsequent stimulation. When the subject pressed a key on the keyboard, all rectangles reverted back to their green colour. After 1 s, the 4‐s stimulation was initiated during which all targets were flickering in accordance with their frequency/phase combination achieved by means of sinusoidally modulating their luminosities (Manyakov et al., 2013). Between trials, the subject was allowed to take shorter breaks and a longer break (±5 min) between sessions. In each session, all targets were cued 15 times in a pseudorandom order. A visualisation of one trial can be seen in Figure 1a.

The experiment was implemented in and presented using MATLAB (2012a) with the Psychophysics Toolbox (version 3) (Kleiner et al., 2007) for precise timing.

### 5.4 Data analysis

#### 5.4.1 Correction of the stimulation profile

As the commercial laptop monitor used in the experiment did not have a linear gamma profile, the sinusoidally sampled luminosity profile requested in the implementation did not actually reflect a sinusoidal profile to the subject. Therefore, prior to analysis, the sinusoidal stimulation profile was corrected to reflect the actual stimulation profile presented to the subject. This was done with the help of a Chroma Meter CS‐100A (Konica Minolta, Belgium) to measure the luminosity output level of the monitor for each unique requested intensity level. Using the obtained gamma curve (Supplementary Figure S1a), the sinusoidal stimulation profile was then corrected and rescaled in the range from −1 to 1 (Supplementary Figure S1b).

#### 5.4.2 Pre‐processing

The raw ECoG data were re‐referenced offline to the common average reference and cut into 6 s epochs from 1 s prior to stimulation onset until 1 s after the stimulation offset. Each epoch was then labelled with the frequency and phase of the gazed (foveal) target. In total, 360 (=90 × 4) labelled epochs were extracted for further analysis.

#### 5.4.3 Extraction of the fundamental response

As the SSVEP is characterised by a prominent peak at the fundamental frequency, analysis typically focuses on a narrow stimulus‐dependent band‐pass filter around this frequency. However, to avoid potential filter‐artefacts (e.g., phase distortion) introduced by selective filtering for each epoch, in this work, all epochs were filtered with an identical fourth‐order zero‐phase Butterworth band‐pass filter with cut‐off frequencies of 10 and 16 Hz, determined by the (Fourier) frequency spectrum that exhibited a pronounced increase within these bounds compared to the spectrum of pre‐ and post‐stimulus signals (Supplementary Figure S2). It is worth noting that, even though the zero‐phase filter temporally smears out the amplitude of the signal (de Cheveigné & Nelken, 2019), maintaining the correct phase information is crucial for our analysis. From the filtered epochs, the analytic amplitude and phase of each epoch were then obtained as the real and imaginary part of the Hilbert transform respectively.

#### 5.4.4 Phase locking

Traditionally, phase locking is used to investigate functional connectivity by assessing whether neural responses at two distinct spatial locations are in synchronisation within a given frequency band. However, in this study, the phase of the fundamental component, extracted using the procedure detailed above, was analysed with regard to the phase of the stimulation profile presented to the subject. The phase of the stimulation profile was obtained by the imaginary part of the Hilbert transformed gamma‐corrected sinusoidal profile in which the original stimulation profile was given by 2*πft* + *ϕ*, where *f* is the frequency of the gazed target, *ϕ* is the phase of the gazed target and *t* ∈ [−1, 5] is the stimulation time sampled at the sampling rate of the epochs.

Using the temporal phase traces of the stimulus and the fundamental response, phase locking for each epoch can be calculated by subtracting the latter from the former and by projecting the resultant phase difference for each sample on the unit circle (Lachaux, Rodriguez, Martinerie, & Varela, 1999):

(1) $${\mathrm{PL}}_{t}=\exp\left(j\Phi_{t}\right),$$

where Φ_*t*_ is the phase difference between the stimulus and the fundamental response at time *t*, and *j* is the imaginary number. The PLV can then be determined as (Lachaux et al., 1999):

(2) $${\mathrm{PLV}}_{t}=\frac{1}{N}|\sum_{t=t_{1}}^{t_{2}}{\mathrm{PL}}_{t}|,$$

where *t*_1_ and *t*_2_ are the indices of the first and last time sample to be included and *N* is the total number averaged samples. For the pre‐ and post‐stimulation PLVs, only the samples with *t* < 0 and *t* > 4 were considered, respectively, and for the phase locking during the stimulation the samples in the interval between 0 and 4 s: *t* ∈ [0, 4]. The phase locking angle is then given by the phase of the averaged vector in Equation (2).

The circular‐linear correlation between the phase locking angles and the gazed frequency was estimated using the approach described in (Kempter, Leibold, Buzsáki, Diba, & Schmidt, 2012); the Watson–Williams test was performed using the CircStat toolbox (Berens, 2009).

#### 5.4.5 Calculation of the modulogram

To identify other components that synchronise with the stimulation, a phase‐amplitude coupling analysis was performed. For each epoch, the instantaneous phase of the corresponding gazed stimulus was extracted using the procedure described above. The amplitude of the neural response was extracted by filtering the epoch between *f* − 2.5 and *f* + 2.5Hz using a fourth‐order zero‐phase Butterworth filter followed by the extraction of the instantaneous amplitude using the Hilbert transform. In line with the procedure described in (Tort, Komorowski, Eichenbaum, & Kopell, 2010), the extracted stimulus phase and response amplitude were then used to calculate the MI for the epoch under consideration. Briefly, the phase signal was binned in increments of 5°; the corresponding amplitude signal was averaged to obtain an average amplitude per phase bin, which was then normalised to a unit vector. An adaptation of the Kullback–Leibler distance subsequently yielded the MI:

(3) $$\mathrm{MI}=\frac{\log\left(N\right)+\sum_{j=1}^{N}P\left(j\right)\log\left[P\left(j\right)\right]}{\log\left(N\right)},$$

where *N* is the number of phase bins and *P*(*j*) is the normalised average amplitude in phase bin *j*. The modulation indices of the epochs during which the same frequency was gazed were then averaged and the centre frequency *f* was varied from 3 to 150 Hz in steps of 2 Hz to obtain the modulogram shown in Figure 2a.

#### 5.4.6 Extraction of the high‐gamma response

To extract the amplitude of the gamma band for each trial, a similar procedure as for the fundamental response was used. First, the filtering ranges were obtained in a data‐driven manner by calculating and plotting the modulograms with the help of the procedure described in the previous section. Each re‐referenced epoch was then band‐pass filtered between 55 and 125 Hz by means of a fourth‐order zero‐phase Butterworth filter followed by Hilbert transform to obtain the instantaneous amplitude.

The 95% confidence interval for each time point, as shown by the shaded area in Figure 2c and Supplementary Figure S6, was obtained from 1,000 bootstrapped samples and calculated using the bias‐corrected and accelerated approach. The data points in each bootstrapped sample were sampled with replacement from the instantaneous high‐gamma amplitudes of all epochs labelled with the given frequency and zero phase.

#### 5.4.7 Phase‐amplitude coupling

The coupling between the stimulation and the high‐gamma amplitude was quantified using phase‐amplitude coupling. First, for each epoch, the phase of the stimulation and the instantaneous amplitude of the high‐gamma response epoch were extracted using the procedures described above. The coupling between the two traces was then determined by calculating the MI as described above. The modulation indices were grouped per gazed frequency and a surrogate group was included to obtain a baseline on the MI for non‐coupled signals. The surrogate group consisted of the instantaneous high‐gamma amplitude traces of 1,000 random epochs, selected (with replacement) from all the epochs. Each of those amplitude traces was then matched with the phase trace of the stimulation profile of a randomly selected epoch after which the MI was calculated. Since the Lilliefors test showed that the modulation indices of the groups were not consistently normally distributed, we used the Wilcoxon rank‐sum test to compare the distributions of the modulation indices for each of the five stimulation frequencies and the surrogate group to each other.

#### 5.4.8 Fundamental and high‐gamma latency

The fundamental, high‐gamma and internal latencies were measured in the time‐domain as the time‐interval between the peaks of the corresponding components (i.e., stimulation profile and fundamental amplitude, stimulation profile and instantaneous high‐gamma amplitude, and instantaneous high‐gamma amplitude and fundamental amplitude, respectively).

First, for each epoch, the time‐domain averages (Luo & Sullivan, 2010; Wittevrongel, Khachatryan, Fahimi Hnazaee, Camarrone, et al., 2018; Wittevrongel & van Hulle, 2017) of the two components were obtained by cutting both components into consecutive overlapping segments whose length equals three periods of the corresponding stimulation frequency. The first sample of each segment (i.e., the cutting points) was given by multiples of one period of the gazed frequency. Next, the peaks of both time‐domain averages were determined using the *findpeaks* function in MATLAB with a minimum inter‐peak distance set to 75% of the period of the gazed frequency to make the procedure more robust. As the coupling or phase locking was already established prior to the latency analyses, this parameter does not bias the measurement. As the components are periodic in nature, multiple peak‐to‐peak latencies can be obtained: the reported latencies were the smallest ones that exceeded a predefined latency. For the fundamental component, the minimal latency was set to 80 ms as previous studies have reported estimated fundamental latency of 100–150 ms. For the high‐gamma latencies, the minimal latency was set to 30 ms based on previous literature that reported the earliest responses in V1 around 40 ms. For the internal latencies, no minimal latency was set.

#### 5.4.9 Regression analysis

The relationship between the different latency measurements was assessed using linear regression models. As the different groups have different mean values, the latencies were z‐scored per group (i.e., gazed frequency) prior to fitting, allowing them to be fit in a single model. To avoid large effects of a few outlier trials on the measured relationship between the three latency measures, a robust linear regression analysis was performed with a bisquare weight function.

A similar procedure was used to obtain the regression model between the fundamental latency and the phase locking angle shown in Supplementary Figure S4b. However, as the phase locking angle is a circular measurement, its z‐scoring was performed using the circular mean and circular *SD* provided by the CircStat toolbox (Berens, 2009).

### 5.5 Simulation

To evaluate the presence of spurious phase‐amplitude coupling, we compared our results with those obtained from a simulation in which we used artificially generated triangular/sawtooth signals. For each trial, a sawtooth signal at the corresponding stimulation frequency was created that reached its maximal value at 70% of each period. These signals were then fed into the same phase‐amplitude coupling analysis as used for the neural signals. In both cases, the fundamental phase was obtained by filtering the signals between 10 and 16 Hz, applying the Hilbert transform and extracting the instantaneous phase. For the high‐gamma amplitude, the signals were filtered between 55 and 125 Hz, after which the Hilbert transform was applied and the instantaneous amplitude extracted. The coupling between the phase and amplitudes followed the same procedure as described in Section 5.4.5.

One of the characteristics we compared between the neural and simulated signals was the time during which the gamma amplitude increases. To obtain this measurement for a given trial, the high‐gamma amplitude was extracted using the procedure described above. From the amplitude trace, the time‐domain average was obtained using the same procedure as described in Section 5.4.8. From the average segment, the gamma rising time was given as the time between the minimal and subsequent maximal gamma amplitude. This procedure was repeated for each trial and the results were summarised per stimulation frequency.

## CONFLICT OF INTEREST

The authors declare no conflict of interest.

## AUTHOR CONTRIBUTIONS

Benjamin Wittevrongel and Elvira Khachatryan designed the experiment; Benjamin Wittevrongel, Elvira Khachatryan, Evelien Carrette, Paul Boon, Alfred Meurs, and Dirk Van Roost collected the data; Benjamin Wittevrongel performed the analysis; Benjamin Wittevrongel, Elvira Khachatryan, and Marc M. Van Hulle interpreted the results and wrote the manuscript.

## ETHICS STATEMENT

Prior to participating, the patient was informed about the aim of our study, the experimental procedure and what would be done with the recorded data to which he gave his written consent.

## Supporting information


**Figure S1** (A) Relationship between requested stimulus intensity and stimulus luminance rendered by the laptop monitor used during the experiment. (B) Requested sinusoidal stimulation profile (green line) and gamma‐corrected stimulation profile used in the analysis (orange line).
**Figure S2** (A) Average frequency spectrum for each of the five stimulation frequencies at sub‐dural channel 36 (i.e. the channel with the highest average phase locking value). Harmonic components are visible up to 200 Hz. The black trace represents the baseline frequency spectrum (i.e. before the stimulation started). (B) The frequency spectrum averaged over all trials at subdural channel 36 shows distinctive increased power in a narrow band from 10 to 15 Hz (indicated by the shaded area) during stimulation, as well as increased power in a broader high‐frequency range from 50 to 250 Hz.
**Figure S3** Top panel: Subdural channels (A‐I) that exhibit consistent increases in phase locking during stimulation compared to before and after stimulation. Bottom panels (A‐I): Corresponding boxplots of phase locking values obtained for each of the five stimulation frequencies before, during and after visual stimulation.
**Figure S4** (A) Visual rendition of fundamental latency measurement in the time‐domain. Each panel shows the time‐domain average of the fundamental neural response (in color) and the stimulation profile (in black). The shaded area indicates the minimal latency of 80 ms. (B) The measured single‐trial latencies show a strong correlation with the corresponding phase locking angles.
**Figure S5** Average high‐gamma amplitude across all trials. The shaded area indicates the 95%‐confidence interval. The range of samples that exhibit a significant difference compared to the amplitudes at stimulation onset (i.e. at time 0) are indicated at the bottom of the plot. The first significant samples is found at 40 ms after the stimulation onset. Note that there is also rebound activity following the stimulation offset.
**Figure S6** High‐gamma amplitude resonates with the gazed frequency. Each panel shows the gazed stimulation profile and the temporal variation in the high‐gamma amplitude from one second before stimulus onset until one second after stimulation for subdural channel 36 (channel D is Supplementary Figure S2). Shaded areas indicate the 95%‐confidence interval.
**Figure S7** (A) Average phase‐amplitude coupling plots. Each panel shows the average amplitude of the high‐gamma response (indicated by the length of the bars) with respect to the phase of the stimulus (indicated by the radial angle). (B) Phase of the stimulation at which the high‐gamma amplitude is maximal. Each dot indicates one trial.
**Figure S8** Visual rendition of the high‐gamma latency measurement in the time‐domain. Each panel shows the time‐domain average of the high‐gamma amplitude (in color) and the stimulation profile (in black). The shaded area indicates the minimal latency of 30 ms.
**Figure S9** A coupling analysis between the phase of the fundamental component and the amplitude of the high‐gamma component at subdural channel 36 reveals that the phase angle at which the high‐gamma amplitude is maximal exhibits a high degree of consistency across trials of the same gazed frequency as well as a clear negative trend across the five frequencies. Each dot indicates one trial.
**Figure S10** Each panel shows the time‐domain average of the high‐gamma amplitude (in color) and the fundamental response (in black). In contrast to previous latency measures (cf. Supplementary Figures S3A and S6), no threshold for the minimal latency is defined.
**Figure S11** Regression analysis between (A) the fundamental and internal latencies (B) the high‐gamma and fundamental latencies.

## Data Availability

The dataset supporting the conclusions of this article is not publicly available due to the sensitive nature of the data. Any requests for obtaining the dataset can be directed toward co‐author E. C. (evelien.carrette@ugent.be).
