# Supplementary material for: High‐gamma oscillations precede visual steady‐state responses: A human electrocorticography study
Source: Hum Brain Mapp. 2020 Sep 4;41(18):5341–55. doi: 10.1002/hbm.25196 (PMC7670637; doi:10.1002/hbm.25196)
Supplement: Supplementary file 1 — Figure S1 (A) Relationship between requested stimulus intensity and stimulus luminance rendered by the laptop monitor used during the experiment. (B) Requested sinusoidal stimulation profile (green line) and gamma‐corrected stimulation profile used in the analysis (orange line). Figure S2 (A) Average frequency spectrum for each of the five stimulation frequencies at sub‐dural channel 36 (i.e. the channel with the highest average phase locking value). Harmonic components are visible up to 200 Hz. The black trace represents the baseline frequency spectrum (i.e. before the stimulation started). (B) The frequency spectrum averaged over all trials at subdural channel 36 shows distinctive increased power in a narrow band from 10 to 15 Hz (indicated by the shaded area) during stimulation, as well as increased power in a broader high‐frequency range from 50 to 250 Hz. Figure S3 Top panel: Subdural channels (A‐I) that exhibit consistent increases in phase locking during stimulation compared to before and after stimulation. Bottom panels (A‐I): Corresponding boxplots of phase locking values obtained for each of the five stimulation frequencies before, during and after visual stimulation. Figure S4 (A) Visual rendition of fundamental latency measurement in the time‐domain. Each panel shows the time‐domain average of the fundamental neural response (in color) and the stimulation profile (in black). The shaded area indicates the minimal latency of 80 ms. (B) The measured single‐trial latencies show a strong correlation with the corresponding phase locking angles. Figure S5 Average high‐gamma amplitude across all trials. The shaded area indicates the 95%‐confidence interval. The range of samples that exhibit a significant difference compared to the amplitudes at stimulation onset (i.e. at time 0) are indicated at the bottom of the plot. The first significant samples is found at 40 ms after the stimulation onset. Note that there is also rebound activity following the stimulation o [file HBM-41-5341-s001.pdf]

High-gamma oscillations precede visual steady state responses: a human electrocorticography study Supplementary information

Benjamin Wittevrongel[1]∗, Elvira Khachatryan[1]∗, Evelien Carrette[2], Paul Boon[2], Alfred Meurs[2] Dirk Van Roost[3], Marc M. Van Hulle[1]

[1] Laboratory for Neuro- and Psychophysiology, KU Leuven, Leuven, Belgium
[2] Laboratory of Clinical and Experimental Neurophysiology, Ghent University Hospital, Ghent, Belgium
[3] Department of Neurosurgery, Ghent University Hospital, Ghent, Belgium
[*] corresponding author

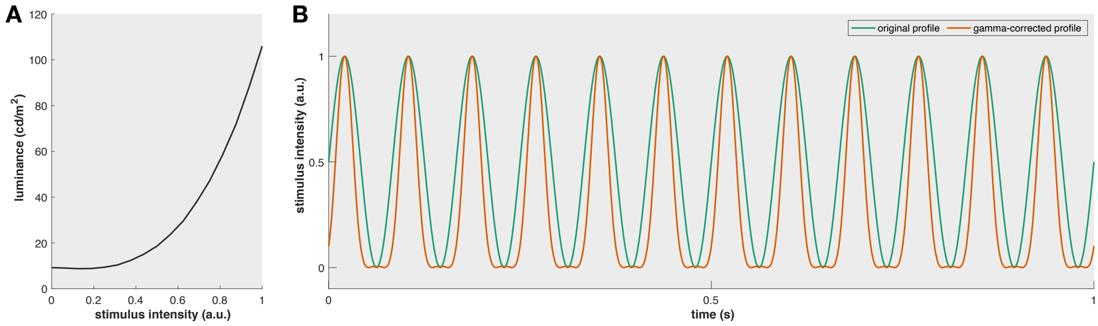

Figure S1: (A) Relationship between requested stimulus intensity and stimulus luminance rendered by the laptop monitor used during the experiment. (B) Requested sinusoidal stimulation profile (green line) and gamma-corrected stimulation profile used in the analysis (orange line).

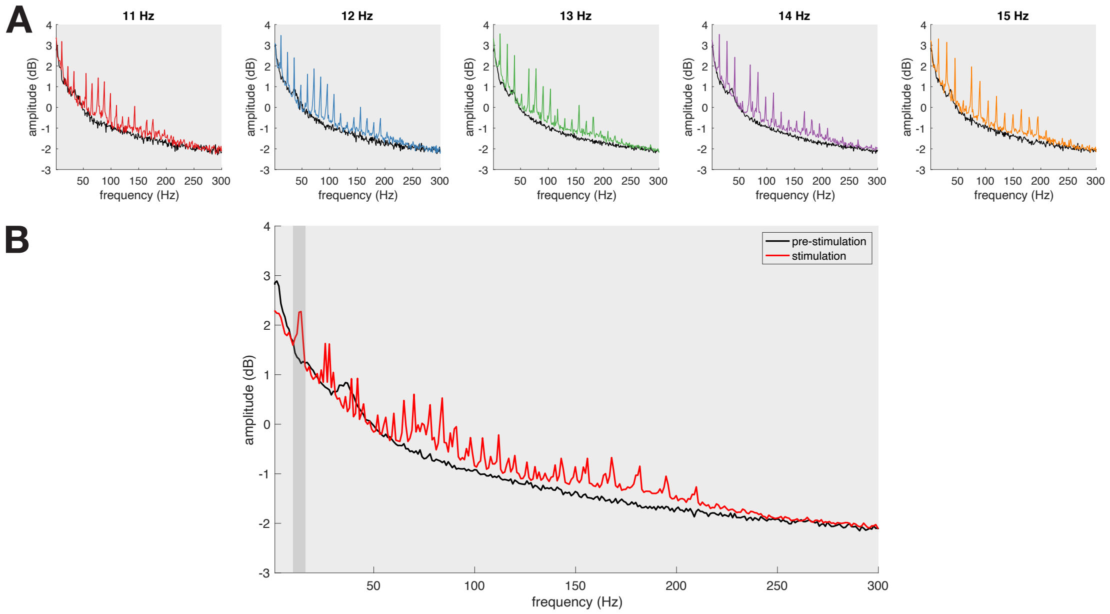

Figure S2: (A) Average frequency spectrum for each of the five stimulation frequencies at subdural channel 36 (i.e. the channel with the highest average phase locking value). Harmonic components are visible up to 200 Hz. The black trace represents the baseline frequency spectrum (i.e. before the stimulation started). (B) The frequency spectrum averaged over all trials at subdural channel 36 shows distinctive increased power in a narrow band from 10 to 15 Hz (indicated by the shaded area) during stimulation, as well as increased power in a broader high-frequency range from 50 to 250 Hz.

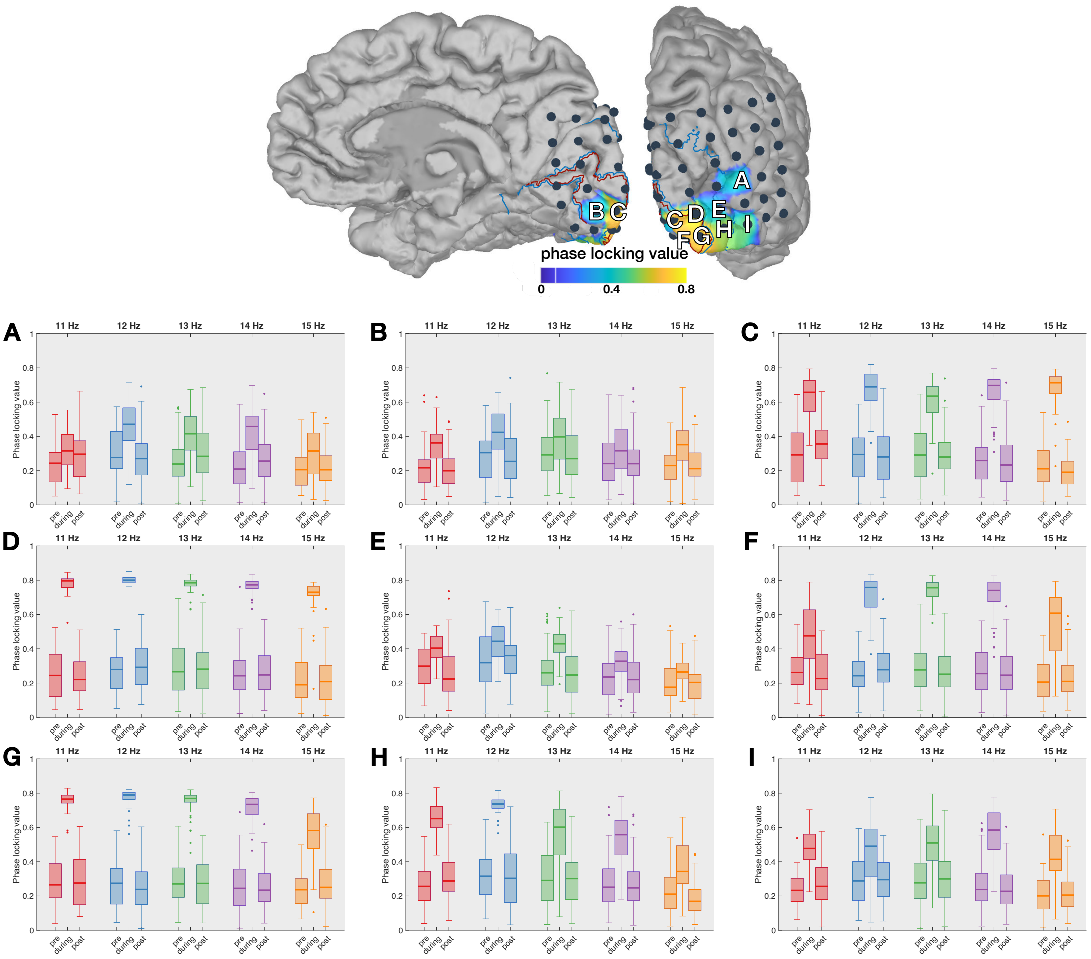

Figure S3: Top panel: Subdural channels (A-I) that exhibit consistent increases in phase locking during stimulation compared to before and after stimulation. Bottom panels (A-I): Corresponding boxplots of phase locking values obtained for each of the five stimulation frequencies before, during and after visual stimulation.

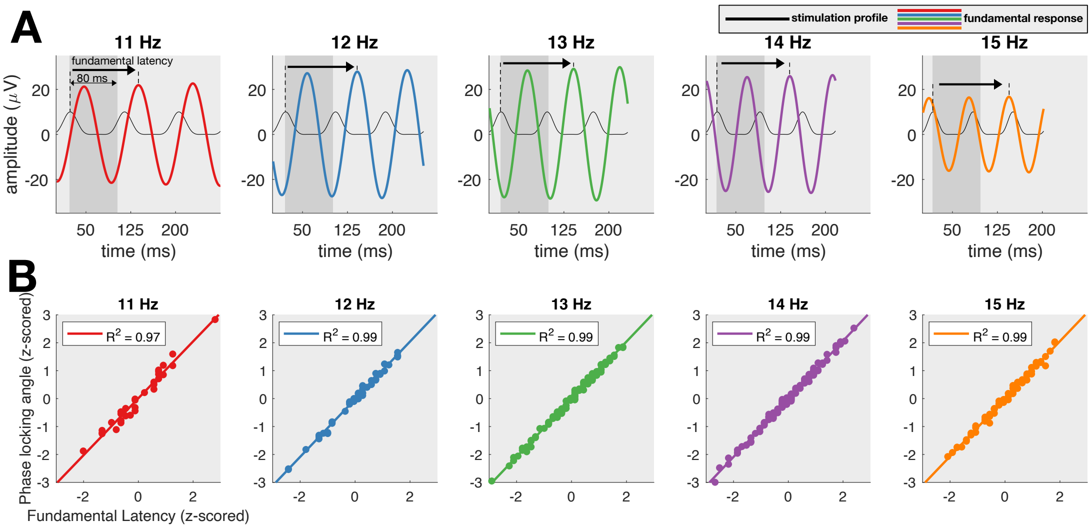

Figure S4: (A) Visual rendition of fundamental latency measurement in the time-domain. Each panel shows the time-domain average of the fundamental neural response (in color) and the stimulation profile (in black). The shaded area indicates the minimal latency of 80 ms. (B) The measured single-trial latencies show a strong correlation with the corresponding phase locking angles.

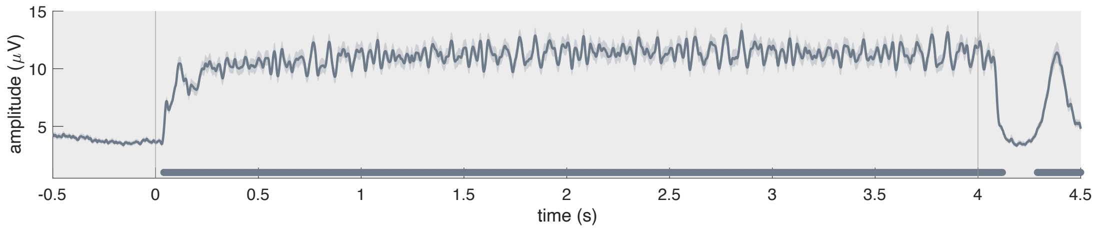

Figure S5: Average high-gamma amplitude across all trials. The shaded area indicates the 95%-confidence interval. The range of samples that exhibit a significant difference compared to the amplitudes at stimulation onset (i.e. at time 0) are indicated at the bottom of the plot. The first significant samples is found at 40 ms after the stimulation onset. Note that there is also rebound activity following the stimulation offset.

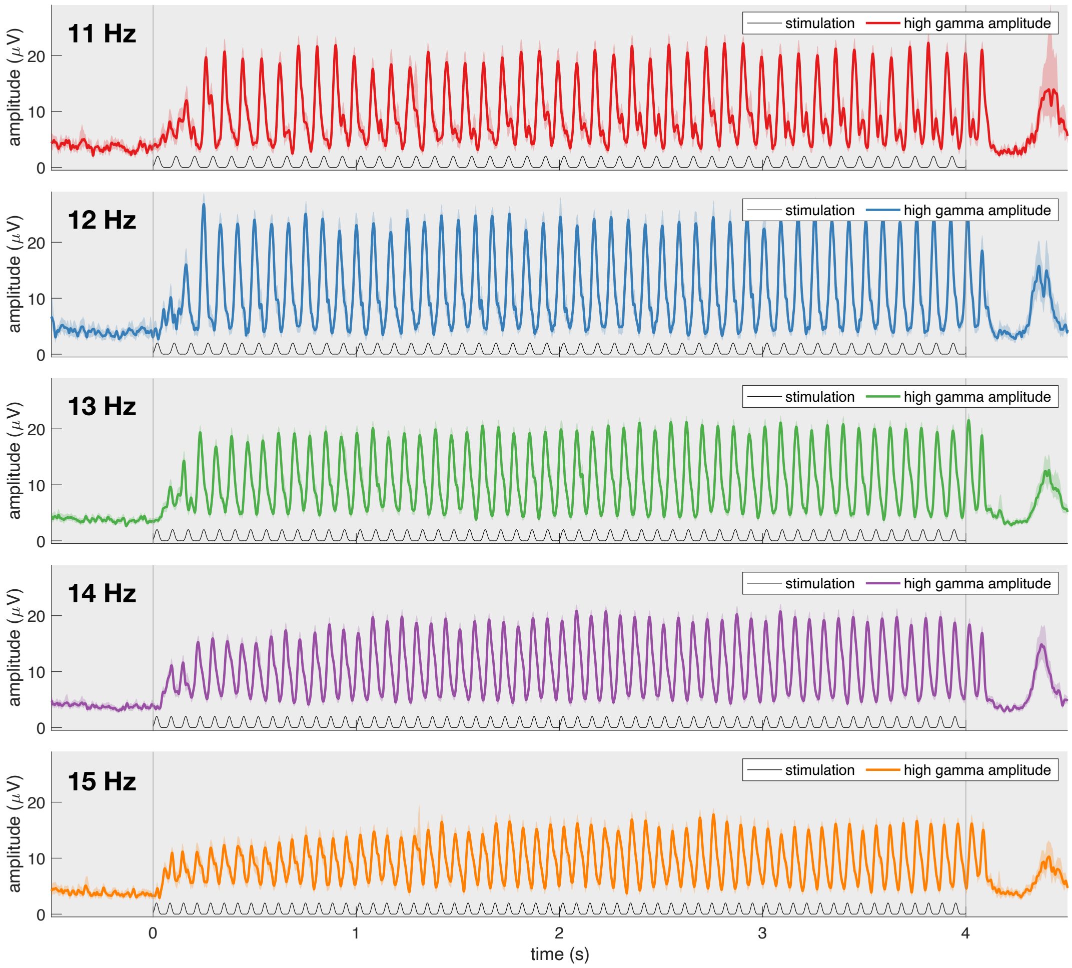

Figure S6: High-gamma amplitude resonates with the gazed frequency. Each panel shows the gazed stimulation profile and the temporal variation in the high-gamma amplitude from one second before stimulus onset until one second after stimulation for subdural channel 36 (channel D is Supplementary figure S2). Shaded areas indicate the 95%-confidence interval.

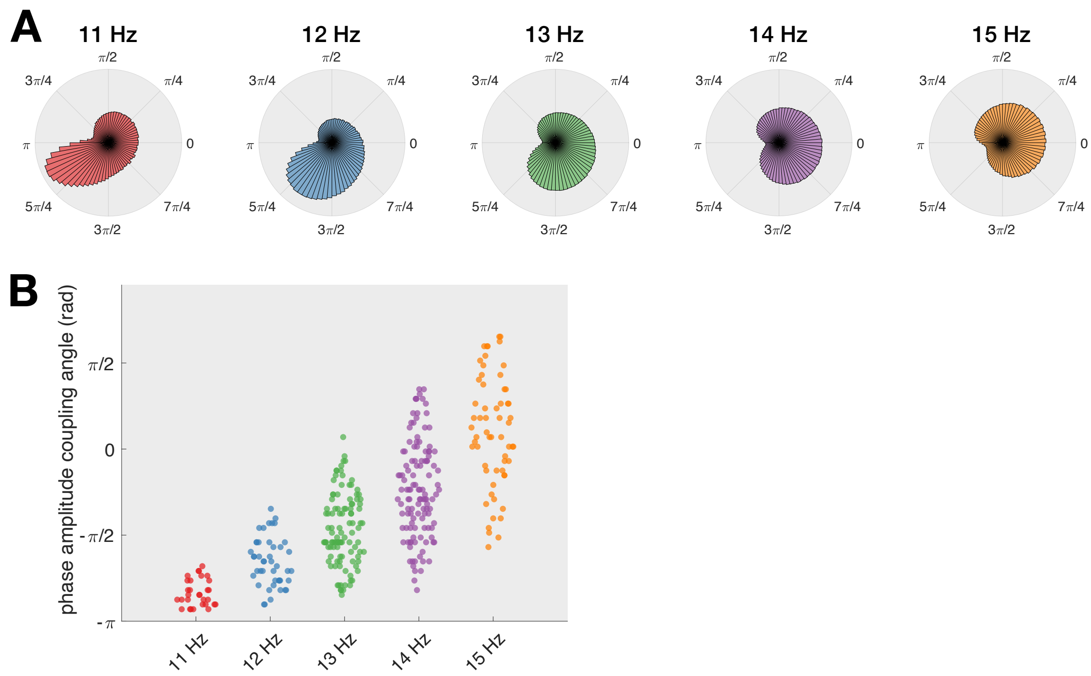

Figure S7: (A) Average phase-amplitude coupling plots. Each panel shows the average amplitude of the high-gamma response (indicated by the length of the bars) with respect to the phase of the stimulus (indicated by the radial angle). (B) Phase of the stimulation at which the high-gamma amplitude is maximal. Each dot indicates one trial.

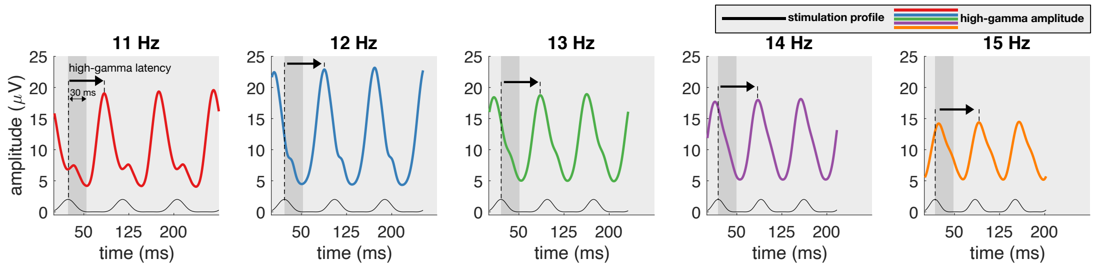

Figure S8: Visual rendition of the high-gamma latency measurement in the time-domain. Each panel shows the time-domain average of the high-gamma amplitude (in color) and the stimulation profile (in black). The shaded area indicates the minimal latency of 30 ms.

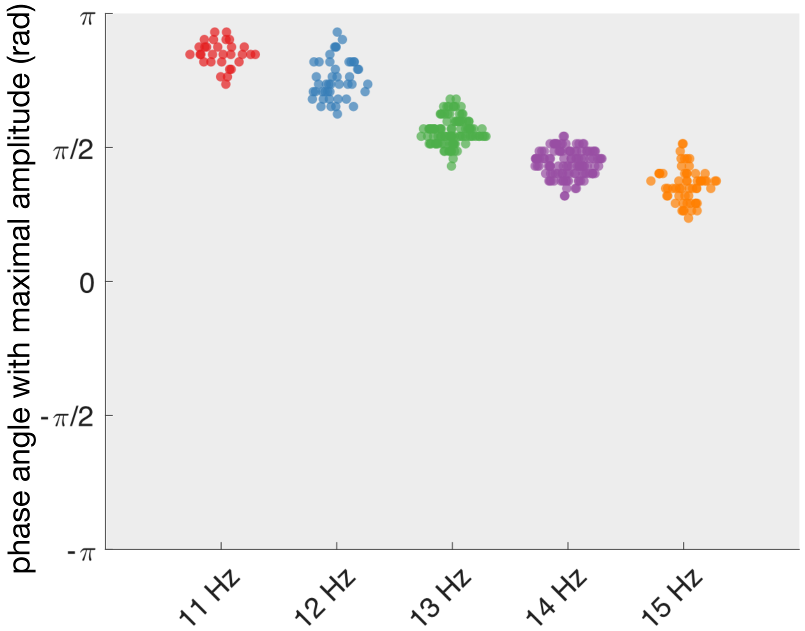

Figure S9: A coupling analysis between the phase of the fundamental component and the amplitude of the high-gamma component at subdural channel 36 reveals that the phase angle at which the high-gamma amplitude is maximal exhibits a high degree of consistency across trials of the same gazed frequency as well as a clear negative trend across the five frequencies. Each dot indicates one trial.

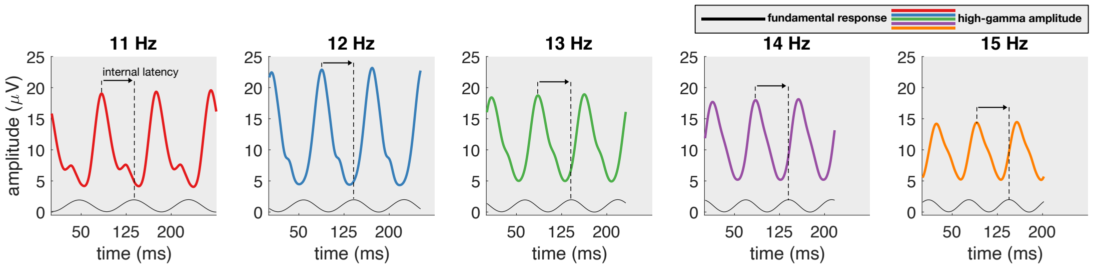

Figure S10: Each panel shows the time-domain average of the high-gamma amplitude (in color) and the fundamental response (in black). In contrast to previous latency measures (cf. Supplementary figures S4A and S8), no threshold for the minimal latency is defined.

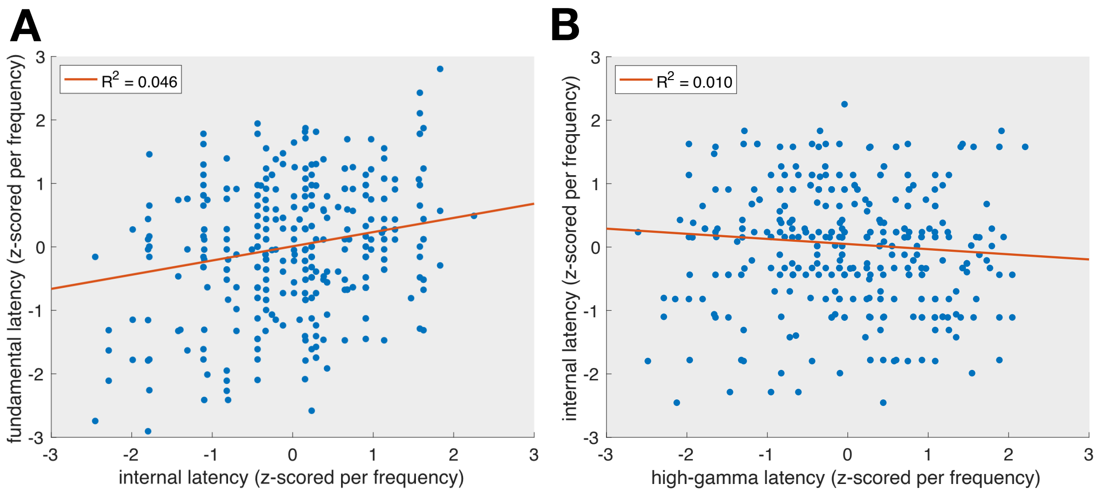

Figure S11: Regression analysis between (A) the fundamental and internal latencies (B) the high-gamma and fundamental latencies.
